# Supplementary figures and images for: An exploratory machine learning study on paediatric abdominal pain phenotyping and prediction
Source: PLoS One. 2025 Nov 5;20(11):e0336215. doi: 10.1371/journal.pone.0336215 (PMC12588484; doi:10.1371/journal.pone.0336215)

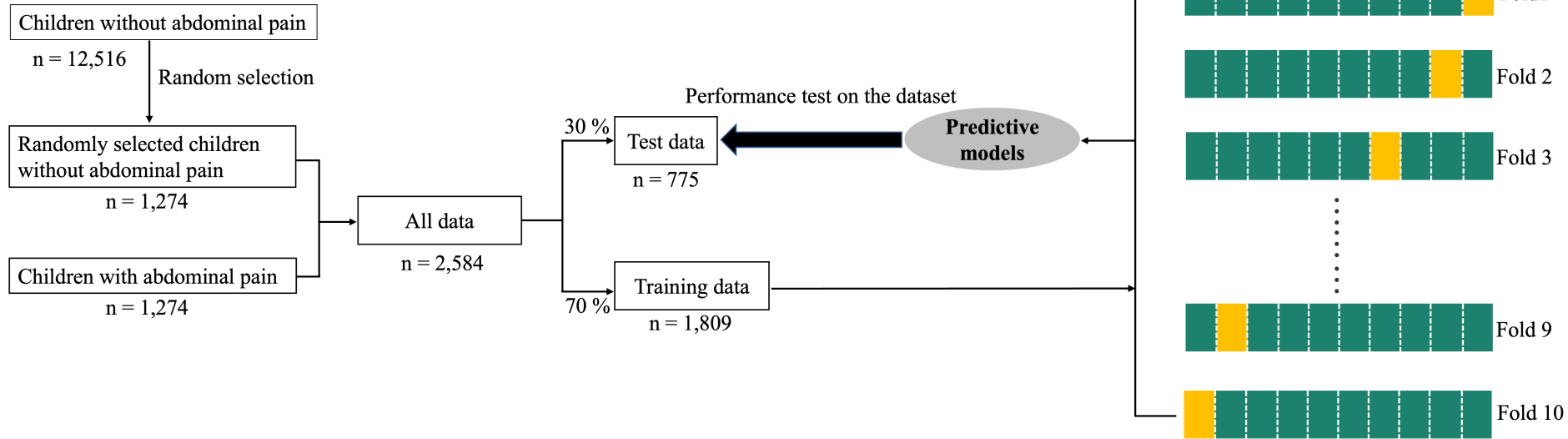

Supplement: S1 Fig — The entire dataset was randomly partitioned into 70% for model training and 30% for out-of-sample testing. Using the training data, we trained predictive models. Grid-search with 10-fold cross-validation was employed to tune the hyperparameters of each model using the training dataset. The training dataset was divided into 10 folds. Nine folds were used as the training dataset, and the remaining one-fold was used as the validation dataset. This process was repeated 10 times to optimize the hyperparameters of each predictive model. The performance of each model was evaluated using the test dataset. (PDF) [file pone.0336215.s001.pdf]
