# Supplementary material for: An exploratory machine learning study on paediatric abdominal pain phenotyping and prediction
Source: PLoS One. 2025 Nov 5;20(11):e0336215. doi: 10.1371/journal.pone.0336215 (PMC12588484; doi:10.1371/journal.pone.0336215)
Supplement: S1 Table — (DOCX) [file pone.0336215.s002.docx]

**S1 Table. Investigated diseases and their corresponding systematized nomenclature of medicine clinical terms (SNOMED-CT) codes**

| **Disease name notation in this study** | **SNOMED-CT** | **SNOMED-CT code** |
| --- | --- | --- |
| Abdominal pain | Abdominal pain | 21522001 |
|  | Generalized abdominal pain | 102614006 |
|  | Abdominal wall pain | 162042000 |
| Allergic diseases | Asthma | 195967001 |
|  | Eczema | 43116000 |
|  | Allergic rhinitis caused by pollen | 21719001 |
|  | Urticaria | 126485001 |
| Appendicitis | Appendicitis | 74400008 |
| Arthritis | Multiple joint pain | 35678005 |
|  | Osteoarthritis | 396275006 |
|  | Lumbar spondylosis | 239880009 |
|  | Cervical osteoarthritis | 387800004 |
| Autism | Autistic disorder | 408856003 |
| Celiac disease | Celiac disease | 396331005 |
| Chronic fatigue syndrome | Chronic fatigue syndrome | 52702003 |
| Chronic muscle pain | Fibromyalgia | 203082005 |
| Constipation | Chronic constipation | 236069009 |
| Depressive disorder, bipolar disorder | Depressive disorder | 35489007 |
|  | Postpartum depression | 58703003 |
|  | Bipolar disorder | 13746004 |
| EDS, JHS | Generalized benign Joint hypermobility | 240261009 |
|  | Hypermobility syndrome | 85551004 |
|  | Ehlers-Danlos Syndrome | 398114001 |
|  | Hypermobile Ehlers-Danlos syndrome | 30652003 |
| FD | Nonulcer dyspepsia | 3696007 |
| GORD | Gastroesophageal reflux disease | 235595009 |
| IBD, colitis | Crohn's disease | 34000006 |
|  | Ulcerative colitis | 64766004 |
|  | Infectious colitis | 39341005 |
|  | Colitis presumed infectious | 79099006 |
|  | Diverticulitis of colon | 111359004 |
| IBS^*^ | Irritable bowel syndrome with diarrhea | 197125005 |
|  | Irritable bowel syndrome characterized by constipation | 440630006 |
|  | Irritable bowel syndrome characterized by alternating bowel habit | 440544005 |
|  | Irritable bowel syndrome | 10743008 |
| Intellectual disability | Intellectual disability | 110359009 |
| Migraine | Migraine | 37796009 |
| obsessive-compulsive disorder | Obsessive-compulsive disorder | 191736004 |
| Schizophrenia | Schizophrenia | 58214004 |
| ^*^IBS status was assessed only in mothers. Given that IBS is defined by the presence of abdominal pain, its inclusion in this study—designed to investigate factors associated with abdominal pain—was considered methodologically inappropriate.  FD, functional dyspepsia; GORD, gastro-oesophageal reflux disease; IBD, inflammatory bowel disease; IBS, irritable bowel syndrome; EDS, Ehlers-Danlos syndrome; JHS, joint hypermobility syndrome; SNOMED-CT, Systematized Nomenclature of Medicine Clinical Terms | | |
