# Supplementary material for: An exploratory machine learning study on paediatric abdominal pain phenotyping and prediction
Source: PLoS One. 2025 Nov 5;20(11):e0336215. doi: 10.1371/journal.pone.0336215 (PMC12588484; doi:10.1371/journal.pone.0336215)
Supplement: S2 Table — (DOCX) [file pone.0336215.s003.docx]

**S2 Table. Hyperparameters of UMAP and HDBSCAN**

| **Hyperparameters of UMAP** |
| --- |
| n_neighbors=15, n_components=3, metric='euclidean', min_dist=0.1, spread=1.0, set_op_mix_ratio=1.0, local_connectivity=1, repulsion_strength=1.0, negative_sample_rate=5, random_state=0 |
| **Hyperparameters of HDBSCAN** |
| min_cluster_size=130, min_samples=None, metric='euclidean', alpha=1.0, cluster_selection_epsilon=0.0, cluster_selection_method='eom', allow_single_cluster=False, prediction_data=False |
| UMAP, Uniform Manifold Approximation and Projection; HDBSCAN, Hierarchical Density-based Spatial Clustering of Applications with Noise |
