# Supplementary material for: An exploratory machine learning study on paediatric abdominal pain phenotyping and prediction
Source: PLoS One. 2025 Nov 5;20(11):e0336215. doi: 10.1371/journal.pone.0336215 (PMC12588484; doi:10.1371/journal.pone.0336215)
Supplement: S3 Table — (DOCX) [file pone.0336215.s004.docx]

**S3 Table. Frequency of variables used in machine learning predictive models**

|  | **Children without abdominal pain**  **(n = 1,274)** | **Children with abdominal pain**  **(n = 1,274)** | ***p* value** |
| --- | --- | --- | --- |
| Female | 590 (46.3) | 659 (51.7) | 0.01 |
| Route of birth, vaginal | 941 (73.9) | 943 (74.0) | 0.96 |
| Pakistani | 485 (45.8) | 703 (58.6) | <0.01 |
| White British | 382 (36.1) | 267 (22.3) | <0.01 |
| Other ethnicities | 191 (18.1) | 229 (19.1) | 0.56 |
| Allergic diseases | 457 (35.9) | 482 (37.8) | 0.32 |
| Appendicitis | 1 (0.1) | 19 (1.5) | <0.01 |
| Constipation | 13 (1.0) | 31 (2.4) | 0.01 |
| GORD | 35 (2.7) | 62 (4.9) | 0.01 |
| Mother’s abdominal pain | 392 (30.8) | 557 (43.7) | <0.01 |
| Mother’s allergic disease | 492 (38.6) | 515 (40.4) | 0.37 |
| Mother’s arthritis | 12 (0.9) | 15 (1.2) | 0.7 |
| Mother’s depressive disorder, bipolar disorder | 274 (21.5) | 252 (19.8) | 0.3 |
| Mother’s FD | 14 (1.1) | 20 (1.6) | 0.39 |
| Mother’s GORD | 83 (6.5) | 102 (8.0) | 0.17 |
| Mother’s IBS | 85 (6.7) | 101 (7.9) | 0.25 |
| Mother’s migraine | 187 (14.7) | 222 (17.4) | 0.07 |
| FD, functional dyspepsia; GORD, gastro-oesophageal reflux disease; IBD, inflammatory bowel disease; IBS, irritable bowel syndrome | | | |
