# Supplementary material for: An exploratory machine learning study on paediatric abdominal pain phenotyping and prediction
Source: PLoS One. 2025 Nov 5;20(11):e0336215. doi: 10.1371/journal.pone.0336215 (PMC12588484; doi:10.1371/journal.pone.0336215)
Supplement: S4 Table — (DOCX) [file pone.0336215.s005.docx]

**S4 Table. Characteristics of all children (N = 13,790)**

| **Background** |  |
| --- | --- |
| Average age at the extraction ± SD (years) | 8.3 ± 1.1 |
| Average age at the diagnosis of abdominal pain ± SD (years) | 5.6 ± 2.7 |
| Gender, female, n (%) | 6,674 (48.4) |
| Route of birth, (vaginal)*, n (%) | 10,358 (76.8) |
| Ethnicity**, n (%) |  |
| Pakistani | 5,453 (47.1) |
| White British | 4,038 (34.9) |
| Other ethnicities | 2,076 (17.9) |
| Abdominal pain, n (%) | 1,274 (9.2) |
| **Frequency of diseases, n (%)** |  |
| Allergic diseases | 5,266 (38.2) |
| Asthma | 1,383 (10.0) |
| Hay fever | 851 (6.2) |
| Urticaria | 695 (5.0) |
| Eczema | 3,828 (27.8) |
| Appendicitis | 51(0.4) |
| Arthritis | 7 (0.1) |
| Celiac disease | 33 (0.2) |
| Constipation | 199 (1.4) |
| FD | 7 (0.1) |
| GORD | 408 (3.0) |
| IBD, colitis | 6 (0.04) |
| Migraine | 69 (0.5) |
| EDS, JHS | 13 (0.1) |
| Autism | 40 (0.3) |
| Intellectual disability | 18 (0.1) |
| Mother’s abdominal pain | 4,616 (33.5) |
| Mother’s allergic disease | 5,540 (40.2) |
| Mother’s appendicitis | 107 (0.8) |
| Mother’s arthritis | 604 (4.4) |
| Mother’s Celiac disease | 87 (0.6) |
| Mother’s chronic fatigue syndrome | 28 (0.2) |
| Mother’s chronic muscle pain | 197 (1.4) |
| Mother’s constipation | 138 (1.0) |
| Mother’s depressive disorder, bipolar disorder | 2,941 (21.3) |
| Mother’s FD | 195 (1.4) |
| Mother’s GORD | 1,047 (7.6) |
| Mother’s IBD | 94 (0.7) |
| Mother’s IBS | 1,003 (7.3) |
| Mother’s migraine | 2,130 (15.4) |
| Mother’s EDS, JHS | 52 (0.4) |
| Mother’s obsessive-compulsive disorder | 71 (0.5) |
| Mother’s schizophrenia | 8 (0.1) |
| Mother’s intellectual disability | 45 (0.3) |
| * 311 values are missing in route of birth. ** 2223 values are missing in ethnicity. | |
| SD, standard deviation; FD, functional dyspepsia; GORD, gastro-oesophageal reflux disease; IBD, inflammatory bowel disease; IBS, irritable bowel syndrome; EDS, Ehlers-Danlos syndrome; JHS, joint hypermobility syndrome | |
