# Supplementary material for: An exploratory machine learning study on paediatric abdominal pain phenotyping and prediction
Source: PLoS One. 2025 Nov 5;20(11):e0336215. doi: 10.1371/journal.pone.0336215 (PMC12588484; doi:10.1371/journal.pone.0336215)
Supplement: S5 Table — (DOCX) [file pone.0336215.s006.docx]

**S5 Table. Non–significant results of logistic regression analysis for the diagnosis of paediatric abdominal pain**

|  | **OR (95% CI)** | ***p* value** |
| --- | --- | --- |
| Gender, female | 0.94 (0.82-1.08) | 0.40 |
| Route of birth, vaginal (vs. caesarean) | 0.93 (0.80-1.09) | 0.36 |
| Arthritis | 21000 (0.00–Inf) | 0.95 |
| FD | 4.01 (0.73-21.90) | 0.11 |
| IBD | 4.00 (0.36-44.20) | 0.26 |
| Autism | 0.89 (0.27-2.89) | 0.84 |
| Intellectual disability | 1.33 (0.22-7.99) | 0.75 |
| EDS, JHS | 8.02 (0.90-71.90) | 0.06 |
| Mother’s appendicitis | 1.87 (0.88-3.98) | 0.11 |
| Mother’s constipation | 1.00 (0.25-4.01) | 1.00 |
| Mother’s Celiac disease | 1.54 (0.74-3.17) | 0.25 |
| Mother’s depressive disorder, bipolar disorder | 1.11 (0.94-1.30) | 0.22 |
| Mother’s IBD | 0.55 (0.27-1.12) | 0.10 |
| Mother’s EDS, JHS | 1.50 (0.63-3.58) | 0.36 |
| Mother’s obsessive-compulsive disorder | 1.00 (0.34-2.93) | 1.00 |
| Mother’s schizophrenia | 1.54 (0.68-3.53) | 0.30 |
| Mother’s intellectual disability | 2.00 (0.13-32.00) | 0.62 |
| IBD, inflammatory bowel disease; EDS, Ehlers-Danlos syndrome; JHS, joint hypermobility syndrome | | |
