# Supplementary material for: An exploratory machine learning study on paediatric abdominal pain phenotyping and prediction
Source: PLoS One. 2025 Nov 5;20(11):e0336215. doi: 10.1371/journal.pone.0336215 (PMC12588484; doi:10.1371/journal.pone.0336215)
Supplement: S6 Table — (DOCX) [file pone.0336215.s007.docx]

**S6 Table. Results of predictive models for paediatric abdominal pain using various machine learning algorithms**

|  | **AUC (95% CI)** | **Accuracy (95% CI)** | **Sensitivity (95% CI)** | **Specificity (95% CI)** | **PPV (95% CI)** | **NPV (95% CI)** |
| --- | --- | --- | --- | --- | --- | --- |
| XGBoost | 0.63 (0.59–0.67) | 0.59 (0.56–0.62) | 0.61 (0.56–0.62) | 0.57 (0.52–0.62) | 0.60 (0.55–0.65) | 0.58 (0.53–0.63) |
| Random Forest | 0.63 (0.59–0.67) | 0.58 (0.55–0.62) | 0.58 (0.53–0.62) | 0.59 (0.54–0.64) | 0.60 (0.55–0.65) | 0.57 (0.52–0.62) |
| CatBoost | 0.67 (0.63–0.71) | 0.62 (0.58–0.65) | 0.68 (0.63–0.73) | 0.55 (0.50–0.61) | 0.62 (0.57–0.66) | 0.62 (0.57–0.68) |
| LightGBM | 0.63 (0.59–0.67) | 0.58 (0.55–0.62) | 0.61 (0.57–0.66) | 0.55 (0.50–0.60) | 0.59 (0.54–0.63) | 0.57 (0.52–0.62) |
| Abbreviations: XGBoost, eXtreme Gradient Boosting; CatBoost, Categorical Boosting; LightGBM, Light Gradient Boosting Machine; CI, confidence interval; PPV, positive predictive value; NPV, negative predictive value; AUC, area under the curve | | | | | | |
